# Editorial Note: Multispacer Sequence Typing Relapsing Fever Borreliae in Africa

**DOI:** 10.1371/journal.pntd.0014414

**Published:** 2026-06-10

**Authors:** The PLOS Neglected Tropical Diseases Editors

The *PLOS Neglected Tropical Diseases* Editors issue this notice to update the previously published Expression of Concern on this article [1,2].

Following the publication of the article and Expression of Concern [1–3], PLOS investigated concerns pertaining to the reported ethical approval and the article’s adherence to *PLOS Neglected Tropical Diseases*’ research ethics policies.

Specifically, the Materials and Methods section of this article [1] reports that the study extracted DNA from blood specimens collected from individuals from Ethiopia in 1994 and 2011, from Tanzania (collection period unknown), and from Senegal in 2010. The ethics approval statement reported in this article states that the study was approved by the Ethics Committee of the Institute Fédératif de Recherche IFR 48, Faculty of Medecine, Marseille, France, but does not report a document reference number. The article does not mention local ethics approval from Ethiopia, Tanzania, or Senegal.

Co-author SJC responded stating that ethics approval for the samples collected in Tanzania was obtained from the Hammersmith Hospitals NHS Trust, England and COSTECH in Dar es Salaam Tanzania. They provided the documents N° NIMR/HQ/R.8a/Vol. VIII/162, N° TBRF/MVH1998/14, N°2001/6073, N° CST/RCA.2001/25/2694/2001, and a document without a reference number for editorial review. Furthermore, SJC stated that ethics approval for the samples collected in Ethiopia in 1994 was obtained prospectively from the research ethics committee of the Faculty of Medicine, Addis Ababa University, but stated that they were unable to retrieve a copy of the original documentation, as this was obtained by another co-author at a different institute.

During the editorial investigation of a seemingly related article [4–6], a representative of the Aix-Marseille Université Ethics Committee stated that samples were not collected during the study and instead the study reused samples previously taken from patients over a long period of time, and they provided documents N° 00.87 MSP/DS/CNERS and N° 001380 MSP/DS/DER for editorial review.

- Document NIMR/HQ/R.8a/Vol. VIII/162 was issued on January 19, 2001, by the National Institute for Medical Research in Tanzania. It provides ethics clearance for a study titled “*Tick-borne Relapsing fever and man – an investigation of persistence of the spirochaete in its human host and existence of alternative animal reservoirs.*”
- Document TBRF/MVH1998/14 was issued on March 5, 1998, by the Diocese of Central Tanganyika Mvumi Hospital. It provides permission to collect blood samples from patients with tick-borne relapsing fever.
- Document 2001/6073 was issued on March 22, 2001, by the Ethics Committee of the Hammersmith Hospital. It provides a 4-year approval for a study titled “*Tick-borne Relapsing fever and man – an investigation of persistence of the spirochaete in its human host and existence of alternative animal reservoirs.*”
- Document CST/RCA.2001/25/2694/2001 was issued on November 26, 2001, by the Tanzania Commission for Science and Technology (COSTECH). It provides a research permit for a study titled “*Tick-borne Relapsing Fever and Man: An investigation of persistence of the spirochaete in its human host and existence of alternative animal reservoirs.*”
- The document without a reference number was issued on March 14, 2001, by the Tanzania Commission for Science and Technology (COSTECH) and provides research clearance for a title titled “*Relapsing Fever and Man – Reservoirs of Borrelia Duttoni in Tanzania*”
- Document N° 00.87 MSP/DS/CNERS is an ethics approval document issued on June 02, 2010 by the Ministère de la Santé et de la Prévention of the Rébublique de Sénégal for protocol SEN37/09 involving a study titled “*“Identification des agents pathogènes responsibles de fièvre au Sénégal (Projet IDEPATH) dans les sites suivants: Niakhar (Fatick), Mlomp (Ziguinchor), Banafassi (Kédougou) et Keur Momar Sarr (Louga).”* It grants a one year approval for a study aiming to identify pathogens responsible for fever at different sites in Senegal.
- Document N° 001380 MSP/DS/DER is an administrative authorization issued on May 31, 2011 by the Ministère de la Santé et de la Prévention of the Rébublique de Sénégal for a study titled “*“Identification des agents pathogènes responsibles de fièvre au Sénégal. Réalisation de tests diagnostiques chez les malades consultant dans les dispensaires de Dielmo, Ndiop, Niakhar, Mlomp, Bandafassi et Keur Momar Sarr”* and grants administrative authorization for one year to implement the studies described in protocols SEN21/09 and SEN37/09.

PLOS reviewed the documentation provided by the institution and concluded that the documents did not fully resolve the journal’s concerns. Specifically,

- The sampling period for the blood samples collected in Tanzania remains unclear. In the absence of this information, the journal is unable to confirm whether the ethics approval documentation meets the journal’s editorial policies on human subjects research.
- It is unclear whether the Senegalese ethics approval documents provided for editorial review cover the full period during which samples were collected from Senegal. In addition, the documents do not mention approval for the secondary analysis of the collected samples as part of a separate study. Furthermore, it is unclear whether this institutional response received for the investigation of [4–6] also applies to this study [1–3].
- The journal has not received prospective ethics approval documentation for the samples collected in Ethiopia.

In light of the unresolved issues, the Expression of Concern stands.
